# Laporte University, Indiana

**Published:** 1845-01

**Authors:** 


### RECORD OF MEDICAL SCIENCE.

*Laporte University, Indiana.*—The Professors in the Medical Department of this University are :—

"Anatomy, Dr. Richards, of St. Charles, Ill.; adj. Prof. Anat., Dr. Shipman, of Cortlandville, N. Y.; Theory and Practice, Dr. Brown, of Kalamazoo, Mich.; Mat. Med., Dr. Knapp, of Chicago, Ill.; Chemistry, Dr. Niles, of Laporte; Surgery, Dr. Meeker, of Laporte; Midwifery, Dr. Hard, of St. Charles, Ill. The Medical Class, the present session, is said to number forty-four.

In a notice of this school, which has been sent to us, it is stated that Professor Hard, of St. Charles, Ill., was at Laporte at the opening of the course, and gave, besides a brilliant introductory, two lectures a day, and that he will return shortly, to go on with and complete his course.—*Bulletin of Med. Science.*

